# Glycosylation of KSHV Encoded vGPCR Functions in Its Signaling and Tumorigenicity

**DOI:** 10.3390/v7041627

**Published:** 2015-03-31

**Authors:** Hui Wu, Liqun Liu, Jun Xiao, Mengdie Chi, Yixiao Qu, Hao Feng

**Affiliations:** 1 Key Laboratory of Protein Chemistry and Developmental Biology of Ministry of Education of China, College of Life Science, Hunan Normal University, Changsha 410081, China; E-Mails: wu2hui218@sina.com.cn (H.W.); zzz54120@hotmail.com (J.X.); chimengdie@163.com (M.C.); 15873156916@163.com (Y.Q.); 2 Division of Pediatric Neurology, Children’s Medical Center, The Second Xiangya Hospital, Central South University, Changsha 410011, China; E-Mail: llq91217@163.com

**Keywords:** KSHV, vGPCR, Glycosylation

## Abstract

Kaposi’s sarcoma-associated herpesvirus (KSHV) is a tumor virus and the etiologic agent of Kaposi’s Sarcoma (KS). KSHV G protein-coupled receptor (vGPCR) is an oncogene that is implicated in malignancies associated with KHSV infection. In this study, we show that vGPCR undergoes extensive N-linked glycosylation within the extracellular domains, specifically asparagines 18, 22, 31 and 202. An immunofluorescence assay demonstrates that N-linked glycosylation are necessary for vGPCR trafficking to the cellular membrane. Employing vGPCR mutants whose glycosylation sites were ablated, we show that these vGPCR mutants failed to activate downstream signaling in cultured cells and were severely impaired to induce tumor formation in the xenograph nude mouse model. These findings support the conclusion that glycosylation is critical for vGPCR tumorigenesis and imply that chemokine regulation at the plasma membrane is crucial for vGPCR mediated signaling.

## 1. Introduction

Kaposi’s sarcoma (KS) was initially described in the late 19th Century as an indo-lent dermal vascular tumor predominantly affecting the lower extremities of elderly Mediterranean men. KS is a tumor which is characterized with abnormal vascular proliferation and the infection of the Kaposi’s sarcoma-associated herpesvirus (KSHV, also known as human herpesvirus 8/HHV-8). KSHV is classified into γ-herpesvirus family and believed to be the etiologic agent of KS and two other B-cell related tumors known as primary effusion lymphoma (PEL) and multicentric Castleman’s disease [1,2]. The KSHV genome encodes an array of proteins that are implicated in modulating host inflammatory responses, angiogenesis and tumor formation, most notably the G protein-coupled receptor (vGPCR or ORF74) [3,4].

KSHV encoded G protein-coupled receptor (vGPCR) is a seven trans-membrane receptor and one of the several known KSHV onco-proteins. vGPCR is constitutively activated and triggers downstream signaling components including the phospholipase C pathway, and PI3 kinase/AKT axis even without ligand binding. It has broad signaling effects *in vitro*, activating NF-κB, NF-AT and AP-1 [5,6,7]. However, chemokine association regulates vGPCR mediated signaling. For example, GRO-a up-regulates and IP-10 down-regulates vGPCR signaling. vGPCR expression *in vivo* caused the tumor formation in nude mice and vGPCR transgenic mice developed disease that resembled human KS lesions [8,9].

vGPCR is a constitutively activated receptor and its expression *ex vivo* triggers a broad spectrum of signaling pathways. Through downstream signaling, this viral oncoprotein promotes the expression of plentiful host and viral functional genes including cytokines, signaling molecules, and transcription factors that result in promoting cell proliferation and endothelial tube formation [10,11,12]. However, vGPCR triggered COS-1 cells to death and caused toxicity in PEL cells when this viral receptor was over-expressed in these cells, which imply that this viral oncogene needs tightly regulated expression and signaling *in vivo* for its function in KSHV pathogenesis. Extensive studies had been done on its signaling and tumorigenecity; however, there were few reports about its post-translational regulation.

vGPCR is predominantly translated from a bi-cistronic mRNA transcript downstream of K14 (vOX2), presumably reducing vGPCR protein expression. Our previous study discovered that KSHV encoded small membrane protein K7 can bind vGPCR through its TM domain and accelerate vGPCR’s degradation by the proteasome through maintaining this onco-protein in the endoplasmic reticulum (ER). Based on this mechanism, K7 dampened vGPCR mediated signaling *ex vivo* and greatly suppressed vGPCR’s tumorigenicity *in vivo* [13]. Our data demonstrated that KSHV has evolved mechanisms such as the posttranslational degradation to achieve a temporary expression of the constitutively active vGPCR during lytic infection.

Posttranslational modification (PTM) is the major regulation of G protein-coupled receptor, such as sulfation, hydroxylation, acylation, *etc.* Actually, the refinement modification of vGPCR is very important for this viral oncoprotein function *in vivo* and *in vitro*. We previously revealed that vGPCR incorporates sulfate groups within its extracellular N-terminal tyrosine residues (Y26 and Y28) and that the tyrosine sulfation is crucial for its tumorigenicity in nude mice although it does not impact vGPCR mediated signaling [14,15].

vGPCR contains four N-X-T/S motifs (asparagine residues 18, 22, 31 and 202) in its extracellular domains, which are predicted to be the potential sites of N-linked glycosylation. In this paper, we demonstrate that vGPCR is modified with N-linked glycosylation at the Asn residues 18, 22, 31 and 202, and all the N-linked glycan chains exist in the extra-cellular domains of this seven membrane-spaning viral protein. Immunofluorescence assay and luciferase report assay verified that glycosylation played important roles in vGPCR trafficking to the cellular membrane and vGPCR mediated signaling transduction. Tumor formation in the xenograph nude mouse model revealed that vGPCR mutants devoid of glycosylation had diminished tumor formation ability *in vivo*. All data supported that vGPCR possesses another posttranslational modification, N-linked glycosylation, which regulates vGPCR-mediated signaling and vGPCR-dependent tumorgenesis.

## 2. Materials and Methods

### 2.1. Cells and Plasmids

HEK293T (293T), HeLa and NIH3T3 cells were obtained from the American Type Culture Collection (ATCC). All the cells were grown in DMEM supplemented with 10% fetal bovine serum, 100 units/mL penicillin and 100 mg/mL streptomycin. pcDNA5/FRT-TO-HA-vGPCR and pCDH-HA-vGPCR-EF-Puro were kept in the lab [15]. vGPCR mutants with single site of N-linked glycosylation (N18, N22, N31, N202), with three sites of glycosylation (N18Q, N22Q, N31Q, N202Q) or without glycosylation site (N0) were site-mutated based on the HA-vGPCR and cloned into pcDNA5/FRT-TO or pCDH-EF-Puro empty vectors (System Bioscience, Dallas, TX, USA) separately. Lenti-virus containing vGPCR or its glycosylation mutants were made as before and NIH3T3 stably expressing vGPCR or its glycosylation mutants were established as previously described [13].

### 2.2. Endo H and PNGase F Digestion

293T cells were transfected with FRT-TO-HA-vGPCR or FRT-TO-HA-vGPCR-N0 separately and transfected cells were harvested at 48 h post-transfection and lysed in lysis buffer (50 mM Tris-Hcl/pH7.4, 150 mM NaCl, 1% NP-40, 5 mM EDTA, 0.05% Tween-20) containing protease inhibitor cocktail (Roche). Whole cell lysates (WCL) were divided into two aliquots, one aliquot digested with Endoglycosidase H (Endo H) or Peptide -*N*-Glycosidase F (PNGase F) according to the instruction of the manufacturer (NEB, Beijing, China); the other aliquot was treated with reaction buffer (without glycosidase) and used as control. Briefly, the sample mixed with the glycoprotein denaturing buffer and denatured at 100 °C for 10 min; then Endo H or PNGase F was added with the G5 or G7 reaction buffer and the samples were incubated at 37 °C for 1 h. The glycosidase digested sample and the control were isolated by 10% SDS-PAGE and examined by immunoblot assay.

### 2.3. Immunoblotting

Immunoblot (IB) assays were performed as previously described [14]. Briefly, the samples were isolated by 10% SDS-PAGE and transferred to PVDF membrane. The membrane was blocked with 3% BSA in TBST and probed with primary antibody. The membrane was incubated with goat anti-mouse IgG peroxidase conjugate or goat anti-rabbit IgG peroxidase conjugate (1:5000) and proteins were detected with ECL system (Bio-Rad, Beijing, China). Alternatively, the membrane was incubated with goat anti-mouse IgG alkaline phosphatase conjugate (1:5000) and proteins were visualized with NBT/BCIP system (Pierce). For the commercial primary antibodies, mouse anti-HA antibody (Sigma, Shanghai, China) was used at the dilution ratio of 1:1000; both mouse anti-phospho-IκBα (Ser32/36, cell signaling) antibody and mouse anti-IκBα antibody (Santa Cruz, Shanghai, China) were diluted at 1:1000.

### 2.4. Luciferase Reporter Assay

The reporter cocktail consists of plasmids expressing fire fly luciferase (50 ng/mL) and β-galactosidase (400ng/mL). The expression of β-galactosidase is driven by a housekeeping glucophosphokinase promoter and the fire fly luciferase expression is under the control of response elements of NF-kB (Luci-NF-κB) or NF-AT (Luci-NFAT) transcription factor. 293T cells (in 6-well plate) were transiently transfected with 2.5 mL of luciferase reporter cocktail, 300ng of FRT-TO-HA-vGPCR or vGPCR glycosylation mutants. The cells were harvested and lysed on ice at 36 h post transfection. Centrifuged supernatant was used to measure luciferase and β-galactosidase activity according to manufacturer’s protocol (Promega, Beijing, China).

### 2.5. Immunofluorescence Microscopy

HeLa cells were transfected with FRT-TO-HA-vGPCR or vGPCR glycosylation mutants. The transfected cells were fixed with paraformaldehyde at 24 h post transfection and permeabilized with Triton X-100 (0.2% in PBS) or not (for detecting plasma membrane protein). After stained with primary and secondary antibodies, cells were analyzed by immunofluorescence microscopy as previously described [13]. For commercial antibodies, mouse monoclonal anti-HA antibody (Sigma) was used at the dilution ratio of 1:400 and Alexa 488-conjugated secondary antibody (Molecular Probes, Shanghai, China) was diluted at 1:1000.

### 2.6. Apoptosis Assay

NIH3T3 cells stably expression vGPCR, its glycosylation mutants (N18, N22, N31, N202, N0) or the empty vector were seeded in the six-well plate (5 × 10^5^/well) and the cells were washed 3 times with PBS 18 h post seeding. The washed cells were cultured in serum-free DMEM or Hank’s Balanced Salt Solution (HBSS) for serum starvation-induced apoptosis. The cell viability was determined by typane blue staining at 24 h (HBSS) or 48 h (DMEM) post starvation treatment as previously described [13].

### 2.7. Tumor Formation Assays in Nude Mice

The animal experiments were undertaken according to the institutional ethical guidelines for animal experiments at the Guangzhou Institute of Medicine and Health, Chinese Academy of Sciences. NIH3T3 cells stably expressing the empty vector (pCDH-EF-Puro) and NIH3T3 cells stably expressing vGPCR or its glycosylation mutants (N18, N22, N31, N202, N0) were injected subcutaneously into the flanks of 3 to 5-week-old mice (athymic, nude/nude) separately. Nude mice were euthanized 3 weeks after inoculation, and the tumor weights were determined as described before [14].

## 3. Results

### 3.1. vGPCR Undergoes N-linked Glycosylation within Its Extracellular Sequences

Glycosylation is one of the major modifications of GPCRs and serves as a major post-translational regulatory mechanism for GPCRs. N-linked glycosylation is a common type of glycosidic bond in which N-linked glycans are almost always attached to the nitrogen atom of an asparagine (Asn) side chain that is present as a part of N-X-S/T consensus sequence.

**Figure 1:**
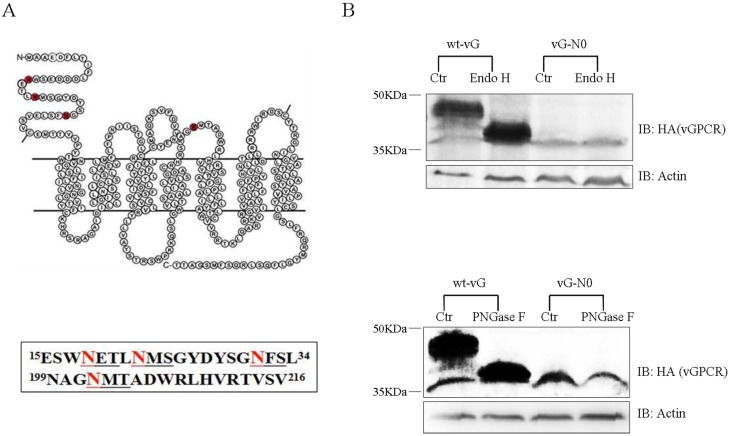
Kaposi’s sarcoma-associated herpesvirus (KSHV) G protein-coupled receptor (vGPCR) undergoes post-translational modification of N-linked glycosylation within its extracellular sequences. (**A**) There are four potential N-linked glycosylation sites in vGPCR. Upper panel: sketch-map of vGPCR and four Asn residues in red (18, 22, 31 and 202); Lower panel: N-X-S/T consensus sequence (underlined) for N-linked glycosylation in vGPCR. (**B**) Glycosidase digestion of vGPCR and vGPCR-N0. HEK293T cells were transfected with FRT-TO-HA-vGPCR or un-glycosylated vGPCR mutant N0, cells were harvested at 48 h posttransfection and lysed, whole cell lysates were digested with Endo H (upper panel) or PGNas F separately. wt-vG: FRT-TO-HA-vGPCR; vG-N0: FRT-TO-HA-vGPCR-N0; Endo H:Endoglycosidase H; PNGase F:Peptide -*N*-Glycosidase F; IB: immunoblot; Ctr:control, the aliquot without glycosidase treatment.

vGPCR contains four Asn residues (18, 22, 31 and 202), which lie within the N-X-S/T consensus site. All the four Asn residues are located within the extracellular sequences of this viral protein: Asn 18, 22 and 31 are located in the N-terminal extracellular motif and Asn 202 is located in the 2nd extracellular loop (Figure 1A). The vGPCR mutant N0, in which all the four Asn (N) residues were mutated into glutamine (Q), was generated and used as the un-glycosylated control. As analyzed by immunoblot assay, the anti-HA antibody detected multiple bands from the whole-cell lysates of HEK293T cells transfected with a plasmid expressing vGPCR. These multiple bands showed that molecular weight of vGPCR varied from ~37 KDa to 50 KDa and the major band was approximately 45 KDa. The major band of vGPCR is larger in molecular weight than the predicted molecular weight (~37 KDa). To test whether these species are glycosylated, we used Endo H and PNGase F to digest and remove glycan chains. While Endo H removes high mannose sugar chain, PNGase F cleaves all glycans, including complex carbohydrates. After digestion with Endo H or PNGase F, there was only one band (~37 KDa) was detected, which indicated that vGPCR was modified with N-linked glycosylation. As expected, digestion of the two glycosidase did not impact the migration of the vGPCR-N0 mutant, which was supposed to have no N-linked glycosylation (Figure 1B). The glycosidase digestion data clearly demonstrates that vGPCR undergoes the modification of N-linked glycosylation.

### 3.2. vGPCR Possesses Four Sites of N-Linked Glycosylation

**Figure 2:**
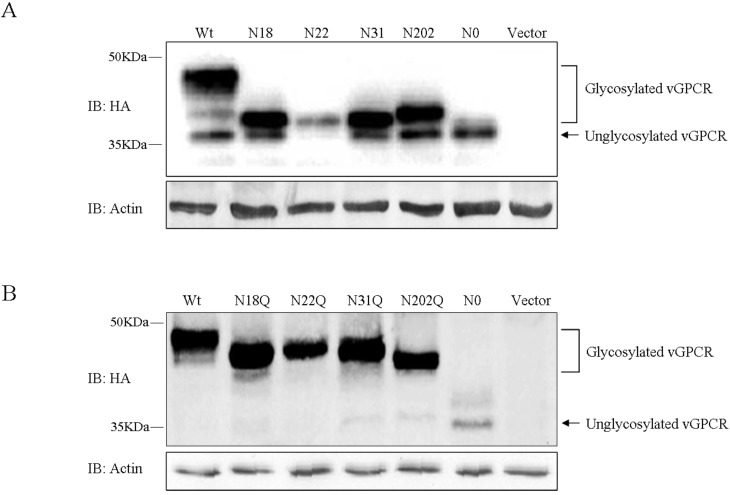
There exist four sites of N-linked glycosylation in vGPCR. (**A**) HEK293T cells were transfected with FRT-TO-HA-vGPCR, un-glycosylated vGPCR mutant N0 or vGPCR mutants with single site of glycosylation (N18, N22, N31 and N202) separately. Transfected cells were harvested at 48 h post-transfection and lysed, whole cell lysates were examined by immunoblot analysis. (**B**) HEK293T cells were transfected with FRT-TO-HA-vGPCR, un-glycosylated vGPCR N0 or vGPCR mutants with three sites of glycosylation (N18Q, N22Q, N31Q and N202Q) separately. Transfected cells were harvested at 48 h post-transfection and lysed, whole cell lysates were examined by immunoblot analysis. Wt: FRT-TO-HA-vGPCR; Vector: FRT-TO empty vector; N18, N22, N31, N202, N18Q, N22Q, N31Q, N22Q, N202Q, N0: FRT-TO plasmids expressing corresponding vGPCR mutants with HA tag.

To exploit which Asn residue (18, 22, 31 and 202) was modified with N-linked glycosylation, vGPCR glycosylation mutants including N18, N22, N31 and N202 were generated, in which three of the four possible glycosylated Asn residues (except the indicated Asn residue) were mutated into glutamine. As shown by immunoblot analysis, all mutants had multiple bands and the major band was around 45 KDa, which migrated more slowly compared with the un-glycosylated N0 mutant (Figure 2A). The data demonstrated that all four Asn residues (18, 22, 31 and 202) are modified with N-linked glycosylation. Accordingly, mutants with three sites of glycosylation (N18Q, N22Q, N31Q and N202Q), in which the indicated Asn residues were mutated into glutamine, migrated more slowly than vGPCR-N0 and slightly faster than wild type vGPCR (Figure 2B). Thes results indicated that each of the four N-X-S/T sites was glycosylated and collectively contributed to the mass increase of vGPCR.

### 3.3. Glycosylation of vGPCR Impacts Its Signal Transduction

**Figure 3:**
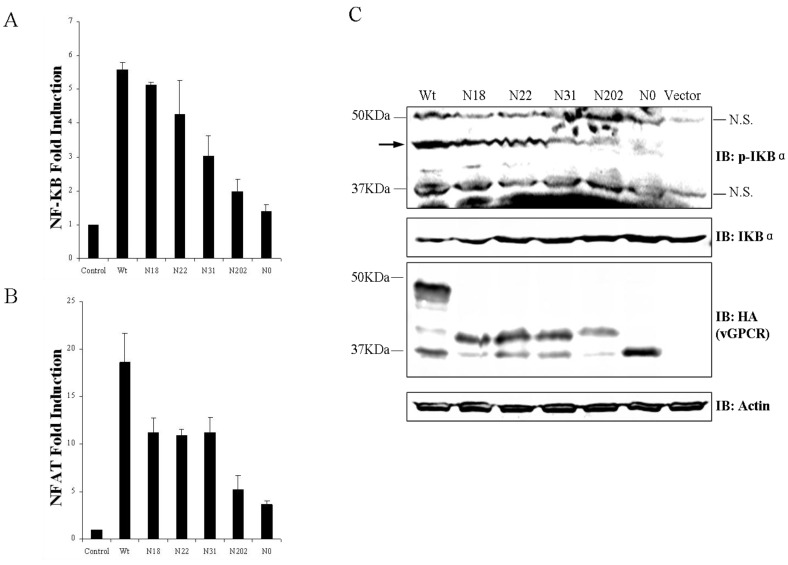
Glycosylation is necessary for vGPCR-mediated signaling. (**A**) and (**B**). Glycosylation is necessary for the induction of NFAT and NF-κB by vGPCR. HEK293T cells were co-transfected with Luci-NF-κB or Luci-NFAT, B-Gal reporter construct, and plasmids expressing vGPCR or vGPCR glycosylation mutants. Transfected cells were harvested and whole cell lysates were used for reporter assay according to the methods. (**A**) NF-κB fold induction by vGPCR and its glycosylation mutants. (**B**) NFAT fold induction by vGPCR and its glycosylation mutants. Data represent the average value of three independent experiments and error bars represent standard deviation. (**C**). Glycosylation is necessary for vGPCR triggered phosphorylation of I-κB. HEK293T cells were transfected with plasmids expressing vGPCR or vGPCR glycosylation mutants separately, Transfected cells were harvested at 24 h post transfection and whole cell lysates were examined by western blot to detect the IκBα phosphorylation. p-IκBα: phospho-IκBα; N.S.: non-specific band; Wt: FRT-TO-HA-vGPCR; Vector: FRT-TO empty vector; N18, N22, N31, N202, N0: FRT-TO plasmids expressing corresponding vGPCR mutants with HA tag; the arrow indicates the phosphorylated IκBα.

Independent of ligand binding, vGPCR is sufficient to activate diverse signaling cascades that culminate in up-regulating cellular gene expression, including those driven by NF-κB and NFAT transcription factors. To probe the role of glycosylation in vGPCR mediated signal transduction, HEK293T cells were transfected with plasmids expressing vGPCR or its glycosylation mutants separately and the transfected cells were applied to luciferase reporter assay at 36 h post transfection. For NF-κB activation, the fold induction by un-glycosylated mutant N0 was less than 20% of that by wild type vGPCR. The NF-κB fold induction by vGPCR mutants with single site of glycosylation (N18, N22, N31 and N022) was decreased more or less (Figure 3A). For NFAT activation, the fold induction by N0 was only 25% of that by wild type vGPCR. The NFAT fold induction by vGPCR mutants with single site of glycosylation (N18, N22, N31 and N022) was decreased more or less (Figure 3B.). To further elucidate the functional mechanism of glycosylation of vGPCR in it mediated NF-κB activation, HEK293T cells were transfected with plasmids expressing vGPCR or its glycosylation mutants separately and the transfected cells were applied to immunoblot assay to examine the I-κB phosphorylation, which reflects NF-κB activation directly. As shown by immunoblot analysis, vGPCR over-expression triggered strong phosphorylation of I-κB alpha (IκB-α), which is an inhibitor of NF-κB (Figure 3C, indicated by the arrow). The phosphorylation of IκB-α triggered by N0 was dramatically attenuated and IκB-α phosphorylation mediated by vGPCR glycosylation mutants (N18, N22, N31 and N022) was decreased more or less (Figure 3C), which matched the NF-κB reporter assay (Figure 3A) very well. All the data demonstrates that N-linked glycosylation of vGPCR is necessary for the activation of NFAT and NF-κB downstream of vGPCR.

### 3.4 Glycosylation of vGPCR Plays an Important Role in Its Membrane Traffic

**Figure 4:**
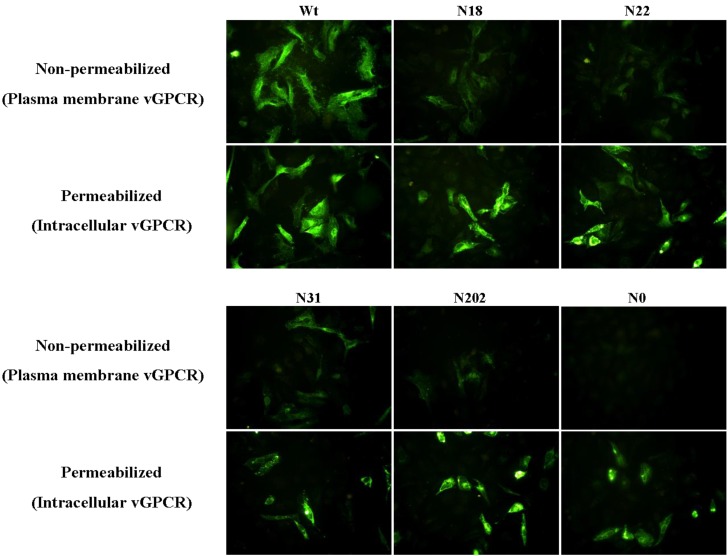
Glycosylation of vGPCR is crucial for its trafficking to plasma membrane. HeLa cells were transfected with plasmids expressing vGPCR or vGPCR glycosylation mutants (N18, N22, N31, N202 or N0) separately and the transfected cells were fixed at 24 h post transfection. The fixed cells were permeabilized (for detecting intracellular vGPCR) or not (for detecting vGPCR on the plasma membrane) and examined by immunoflurescence microscopy as described in methods. WT: FRT-TO-HA-vGPCR; N18, N22, N31, N202, N0: FRT-TO plasmids expressing corresponding vGPCR mutants with HA tag.

Glycosylation is a major regulation factor for the traffic of eukaryotic proteins, especially for GPCR’s membrane traffic. vGPCR is a lytic gene and was detected on the cellular surface of the KSHV latent infected BCBL-1 cells after TPA induction. In addition to the surface presentation, vGPCR resides predominantly in the trans-Golgi network (TGN) when it was expressed in cells [13,14]. To determine whether glycosylation impacts vGPCR’s surface expression, HeLa cells were transfected with plasmids expressing vGPCR or its glycosylation mutants (N18, N22, N31, N202 and N0) separately. The transfected HeLa cells were fixed and analyzed by immunofluorescence (IF) staining. The HeLa cells were permeabilized with Triton-X 100 to detect intracellular vGPCR or without detergent to detect cellular surface vGPCR, in which HeLa cells transfected with plasmid expressing wild type vGPCR as positive control. IF data showed that all the mutants could be detected in the permeabilized HeLa cells. However, vGPCR-N0 was not detected in un-permeabilized HeLa cells while all the mutants with single site of glycosylation (N18, N22, N31, N202) were detected in un-permeabilized HeLa cells. The data showed that vGPCR could not be delivered to cellular surface without the modification of N-linked glycosylation. The mutants with single site of glycosylation (N18, N22, N31 and N202) were delivered to plasma membrane, although their cellular surface expression level was much lower than that of wild type vGPCR. Taken together, the modification of N-linked glycosylation on each of the four Asn residues could help vGPCR traffic to the cellular surface (Figure 4).

### 3.5. Glycosylation of vGPCR Impacts Its Tumorigenicity in Nude Mice

**Figure 5:**
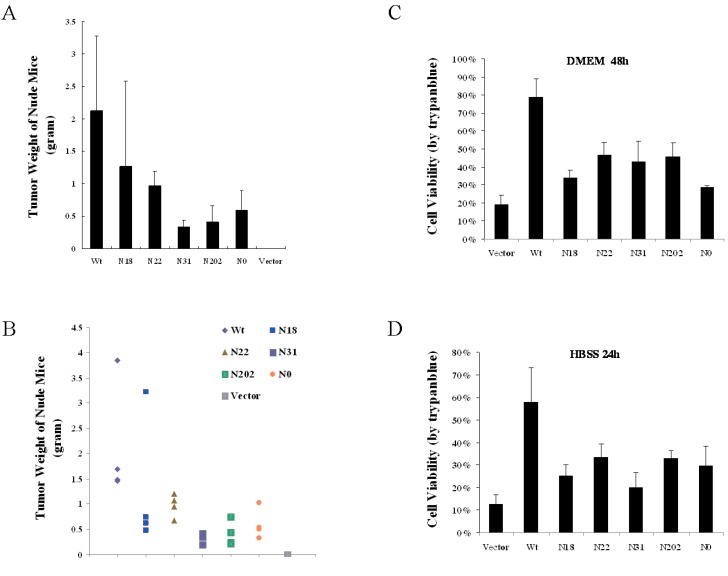
Glycosylation of vGPCR plays a key role in its tumorigenicity. (**A**) and (**B**): Glycosylation is crucial for vGPCR-dependent tumor formation in nude mice. NIH3T3 cells stably expressing vGPCR, its glycosylation mutants (N18, N22, N31, N202, N0) or empty vector (1 × 10^6^ cells/mouse), together with NIH3T3 cells (2 × 10^6^ cells/mouse) were inoculated into the flanks of nude mice separately and nude mice were euthanized at 3 weeks after inoculation. The average tumor weight of each group was shown in (**A**) and the individual tumor weight distribution of each group was shown in (**B**). Data represent 4 independent measurements for each group and error bars denote standard deviation. (**C**) and (**D**): Glycosylation is important for vGPCR-expressing cells’ viability in serum starvation. NIH3T3 cells stably expressing vGPCR or its glycosylation mutants were used for serum induced apoptosis assay according to methods. Data represent three independent assays. DMEM 48 h: serum-free DMEM treatment for 48 h; HBSS 24 h: HBSS treatment for 24 h; Vector: NIH3T3 cells stably expressing the empty vector (pCDH-EF-Puro).

vGPCR possesses tyrosine sulfation in its N-terminal extracellular domain, which is crucial for vGPCR’s tumorigenicity although it is not important for vGPCR mediated signaling. NIH3T3 cells stably expression vGPCR or its glycosylation mutants including N18, N22, N31, N202 and N0 were established and applied to tumor formation assay in nude mice in order to determine whether glycosylation of vGPCR functions in its tumorigenecity. Visual tumors were found in the nude mice inoculated with NIH3T3 cells expressing vGPCR at 10 days post inoculation and visual tumors were found in the nude mice inoculated with NIH3T3 cells expression vGPCR glycosylation mutants at 13~15 days post inoculation separately (data not shown). The nude mice were euthanized 21 days post inoculation and the tumor weight was measured. The average tumor weight of wild type vGPCR group was 2.122 gram; the average tumor weight of N18 group was 1.269 gram, N22 group 0.973 gram, N31 group 0.337 gram and N202 group 0.413 gram. The average tumor weight of N18, N22, N31 and N202 group was 60%, 46%, 16% and 19% of that of the wild type vGPCR group accordingly. Obviously, the tumor formation ability of vGPCR mutants with single site of glycosylation (N18, N22, N31, N202) in nude mice was much decreased compared with that of wild type vGPCR. It was interesting that nude mice of N0 group developed tumor and the average tumor weight was 0.594 gram, which was 28% of that of wild type vGPCR group (Figure 5A,B). To further elucidate the impact of glycosylation on vGPCR mediated tumorigenesis, NIH3T3 cells stably expression vGPCR or its glycosylation mutants (N18, N22, N31, N202 and N0) were applied to apoptosis assay, in which serum-free DMEM or HBSS were used to induce serum starvation-induced apoptosis. NIH3T3 cells expressing wild type vGPCR showed highest viability in both DMEM and HBSS environment after serum deprivation and NIH3T3 cells expressing vGPCR glycosylation mutants (N18, N22, N31, N202 and N0) demonstrated obviously attenuated viability in the condition of serum deprivation, which correlated with their tumorigenesis in nude mice. All the data demonstrates that glycosylation plays an important role in vGPCR mediated tumorigenesis.

## 4. Discussion

KSHV GPCR (vGPCR) is a seven-transmembrane receptor and an acknowledged viral oncogene. vGPCR activates NF-κB and NFAT through downstream signaling and causes tumor formation in mice. Extensive studies have been done on its signaling and tumorigenicity; however, there are few reports about its post-translational modification, such as protein sulfation and protein glycosylation. Glycosylation is a form of co-translational and post-translational modification for eukaryotic proteins and glycans serve a variety of structural and functional roles in membrane and secreted proteins.

vGPCR contains four Asn residues (residues 18, 22, 31 and 202) within its extracellular sequences, which lie within the N-X-S/T consensus sites. Data generated in this paper showed that vGPCR possesses N-linked glycosylation and all the four asparagines in vGPCR are modified with N-linked glycosylation. The glycan chains of vGPCR are all located in its extracellular domain and increase the molecular weight of vGPCR (from ~37 KDa to ~45 KDa). The immunoblot assay showed that N202 mutant migrated most slowly among the mutants with single site of glycosylation (N18, N22, N31 and N202) and N202Q migrated fastest among the mutants with three sites of glycosylation (N18Q, N22Q, N31Q and N202Q), which means the glycan chain on the Asn residue of 202 has the largest molecular weight among the four glycan chains of vGPCR (Figure 2).

Membrane glycoproteins are cotranslationally N-glycosylated in the endoplasmic reticulum (ER) and when properly folded, traffic via the secretory pathway to their final destination such as the plasma membrane. Thus, glycosylation is a key regulator of membrane traffic for many proteins, such as such as Kv1.4, PAR2, SLC4 and SLC26 family [16,17,18]. vGPCR is a lytic gene and was detected on the cellular surface of the KSHV latent infected BCBL-1 cells after TPA induction and vGPCR expressing NIH3T3 cells through immunofluoresent staining [13,14]. The un-glycosylated N0 mutrant was not detected on the cellular surface of transfected HeLa cells, which demonstrates glycosylation is necessary for vGPCR’s traffic to plasma membrane. Any of the four Asn residues with glycosylation is sufficient for vGPCR’s membrane traffic since mutants including N18, N22, N31 and N202 were all detected on the plasma membrane although cellular surface expression level was obviously lower than that of wild type vGPCR (especially N202). The cellular surface expression level of vGPCR mutants with three glycosylation sites (N18Q, N22Q, N31Q and N202Q) was much higher than that of single glycosylation site (N18, N22, N31 and N202), even nearly to that of wild type vGPCR (data not shown here), which implies that cellular surface expression level of vGPCR glycosylation mutants is directly proportional to the number of glycan chains.

vGPCR is a constitutively activated viral receptor and mediates a broad spectrum of signaling pathway; however, ligand association regulates vGPCR mediated signaling, which suggest that cellular surface expression and post-translational modification are crucial vGPCR’s function [19,20]. Actually, our previous data showed vGPCR possesses tyrosine sulfation within its N-terminal extracellular domain. The refinement modification of tyrosine sulfation is specific for vGPCR’s association with GRO-α and crucial for vGPCR-dependent tumorigenesis, although it does not impact vGPCR’s membrane traffic and vGPCR mediated signaling [14]. The data that N0 was not detected on plasma membrane might explain why this un-glycosylated vGPCR mutant is almost deprived of the ability to activate NFAT and NF-κB in the reporter assay. The glycan chains on the site of 18, 22 and 31 are within the extracellular N-terminus of vGPCR, which is the crucial domain for chemokine binding. However, the glycan chain on the site of 202 is located in the 2^rd^ extracellular loop of vGPCR, which might explain why N202 mutant showed the lowest fold induction of NF-KB and NFAT among the four mutants with single glycosyltiaon site (N18, N22, N31 and N202) in reporter assay (Figure 2). All the data emphasize that regulation of vGPCR on the plasma membrane is crucial for the function of this viral G protein-coupled receptor.

The immunoblot assay showed that expression level of N0 mutant in tissue culture was lower than that of wild type vGPCR and other glycosylation mutants when HEK293T cells were transfected with plasmids expressing vGPCR or its glycosylation mutants, which implied the un-glycosylated vGPCR mutant was not as stable as wild type vGPCR. Our data suggests that N-linked glycosylation plays an important role in protein folding and stability of vGPCR. It is imaginable that a N0 mutant, because of being deprived of modification of N-linked glycosylation, was not folded correctly and subjected to being retained in ER instead of being delivered to cellular membrane when this mutant was expressed in tissue culture. Misfolded N0 was targeted for degradation by the proteasome and might cause ER stress during rapid degradation when expressed in tissue culture [21]. That might be the reason why NIH3T3 cells expressing N0 mutant triggered tumor development in nude mice.

In conclusion, this paper demonstrates that vGPCR possesses modification of N-linked glycosylation at the Asn residues of 18, 22, 31 and 202, which is necessary for vGPCR’s membrane traffic. *In vitro* and *in vivo* data elucidate that N-linked glycosylation of vGPCR plays crucial roles in vGPCR mediated signaling and in vGPCR-dependent tumorigenesis.
